# Sexual Orientation and Cervical Cancer Screening Among Cisgender Women

**DOI:** 10.1001/jamanetworkopen.2024.8886

**Published:** 2024-05-06

**Authors:** Kelley Baumann, Hannah Matzke, Caryn E. Peterson, Stacie Geller, Rey Flores, Nikhil G. Prachand, Hunter K. Holt

**Affiliations:** 1 Department of Epidemiology and Biostatistics, University of Illinois Chicago, Chicago; 2 Chicago Department of Public Health, Chicago, Illinois; 3 Center for Research on Women and Gender, University of Illinois Chicago, Chicago; 4 Department of Obstetrics and Gynecology, University of Illinois Chicago, Chicago; 5 Department of Behavioral and Social Health Science, Brown University, Providence, Rhode Island; 6 Department of Family and Community Medicine, University of Illinois Chicago, Chicago

## Abstract

**Importance:**

Lesbian, gay, and bisexual populations face barriers accessing health care in Chicago, Illinois.

**Objective:**

To describe the prevalence of up-to-date cervical cancer screening among lesbian, gay, and bisexual vs heterosexual cisgender women in Chicago.

**Design, Setting, and Participants:**

This retrospective, cross-sectional, population-based study of cisgender women residing in Chicago was completed from 2020 to 2022 using data from the Healthy Chicago Survey, which is conducted annually by the Chicago Department of Public Health. Participants included cisgender women aged 25 to 64 years with no history of hysterectomy. Respondents who self-identified as lesbian, gay, or bisexual or other than straight, lesbian, or bisexual were coded as lesbian, gay, or bisexual (LGB). Respondents who self-identified as straight were coded as heterosexual. Those who reported having a Papanicolaou test within the past 3 years were considered up-to-date with cervical cancer screening. Data analysis was performed from June to October 2023.

**Exposures:**

The primary exposure was sexual orientation. Covariates included age, income level, race, ethnicity, having a primary care practitioner (PCP), and insurance coverage.

**Main Outcomes and Measures:**

Prevalence ratios (PRs), log-based regression models, and interaction analysis were used to describe the association of sexual orientation with up-to-date screening.

**Results:**

The sample included 5167 cisgender women (447 LGB and 4720 heterosexual), aged 25 to 64 years, with no history of hysterectomy. Among LGB cisgender women, 318 (71.14%) reported previous cervical cancer screening compared with 3632 (76.95%) heterosexual cisgender women. The prevalence of up-to-date screening was 10% lower in the LGB group compared with the heterosexual group (PR, 0.90; 95% CI, 0.82-1.00). In regression analysis, having a PCP (PR, 1.43; 95% CI, 1.29-1.59) was associated with up-to-date screening. In interaction analysis, LGB cisgender women with a PCP were 93% more likely to be up-to-date compared with those without a PCP (PR, 1.93; 95% CI, 1.37-2.72).

**Conclusions and Relevance:**

In this cross-sectional study of cervical cancer screening rates between the heterosexual and LGB populations in Chicago, up-to-date cervical cancer screening was associated with having a PCP, regardless of sexual orientation, but this association was greater for LGB individuals. Although LGB populations were less likely to be screened, this disparity may be reduced with more consistent health care access and established care with PCPs.

## Key Points

**Question:**

Are lesbian, gay, and bisexual (LGB) cisgender women less likely than heterosexual cisgender women to be screened for cervical cancer?

**Findings:**

In this cross-sectional study that included 5167 cisgender women in Chicago, Illinois, 71.14% of LGB cisgender women reported up-to-date cervical cancer screening, compared with 76.95% of heterosexual cisgender women. LGB cisgender women with a primary care practitioner (PCP) were 93% more likely to be up-to-date per cervical cancer screening recommendations than LGB cisgender women without a PCP.

**Meaning:**

These findings suggest that improving access to PCPs may reduce the disparity in cervical cancer screening between LGB cisgender women compared with heterosexual cisgender women.

## Teaser

This cross-sectional study describes the prevalence of up-to-date cervical cancer screening among lesbian, gay, and bisexual cisgender women vs heterosexual cisgender women in Chicago, Illinois.

## Introduction

Cervical cancer screening (CCS) in the US has drastically reduced cervical cancer incidence and mortality and is recommended by the US Preventive Services Task Force.^1^ Sixty-four percent of eligible individuals residing in Chicago, Illinois, were screened for cervical cancer from 2021 to 2022.^2^ Systemic racism and discrimination in the health care system, poor CCS insurance coverage, inequitable health care access, and stigma against lesbian, gay, bisexual, transgender, queer, asexual, and intersex (LGBTQAI) individuals have caused CCS services to be underutilized by marginalized populations.^3,4^

LGBTQAI populations face unique barriers to CCS; patients and practitioners often underestimate the risk of contracting high-risk human papillomavirus (HPV) and therefore may not screen as often as recommended.^5,6,7^ Although sexually minoritized populations have poorer health care access in Chicago,^8^ there is limited research on CCS disparities for LGBTQAI patients. This study sought to describe the prevalence of up-to-date CCS among lesbian, gay, and bisexual (LGB) cisgender women compared with heterosexual cisgender women in the US and Chicago in particular. We used the Health Equity Promotion Model, a framework to promote LGBTQAI health that recognizes intersectionality, structural context, and health-promoting and hindering factors, to inform variable selection and interpretation of the data.^9^

## Methods

Data from a retrospective, cross-sectional, population-based study of adults residing in Chicago between 2020 and 2022 were analyzed. Data were collected in the Healthy Chicago Survey (pooled years 2020-2022), an annual survey of approximately 4500 randomly selected adults conducted by the Chicago Department of Public Health (see the eAppendix in Supplement 1).^10^ All data, including race and ethnicity, were self-reported by survey respondents via web or paper interview. This study was approved by the University of Illinois Chicago institutional review board. Participants provided consent through the survey. The data report followed the Strengthening the Reporting of Observational Studies in Epidemiology (STROBE) reporting guidelines for cross-sectional studies.^11^

Variables were identified a priori as confounders (age) and independent variables potentially associated with the CCS outcome (income level,^12^ race,^4^ Hispanic/Latina ethnicity,^4^ having a primary care practitioner [PCP],^13^ and insurance coverage^3,8^) according to their importance in the Health Equity Promotion Model and the relationship between sexual orientation and up-to-date CCS.^9^ Age was included as a confounder because of its association with both identifying with the LGBTQAI community and seeking CCS. Sexual orientation, Hispanic/Latina ethnicity, having a PCP, and insurance coverage are binary variables. Income level categories include less than 199%, 200% to 399%, and greater than or equal to 400% of the Federal Poverty Level. Race categories include Asian American, Pacific Islander or Native Hawaiian, Black or African American, White, or some other race (includes American Indian or Alaska Native).

Respondents who self-identified as lesbian, gay, or bisexual or other than straight, lesbian, or bisexual were coded as LGB. Respondents who self-identified as straight were coded as heterosexual. Up-to-date CCS was measured by the survey question, “How long has it been since your last Pap test?” Those who had a Papanicolaou test within the past 3 years were considered up-to-date. Although this meets current recommendations for CCS, this measure may have underestimated up-to-date CCS for women aged 30 to 64, for whom cotesting with an HPV DNA test every 5 years is commonly recommended and used.^1^ Having a PCP was measured by the survey question, “Do you have at least one person you think of as your personal doctor or health care provider?” The survey did not specify specialty or differentiate between physicians and advanced practice practitioners.

### Statistical Analysis

Data analysis was performed from June to October 2023. Stratified prevalence ratios (PRs), logistic regression models, and interaction analysis were used to describe the association of sexual orientation with up-to-date CCS. Data were weighted using pooled weights to adjust for unequal probability of selection and nonresponse. Interaction was assessed via logistic regression; relative excess proportion due to interaction and a ratio of PRs were calculated according to methods described by Knol et al.^14^ Backward elimination (threshold *P* < .05) was used to identify significant independent variables associated with up-to-date CCS. Variables were retained where at least 1 level met this threshold. Data analysis was performed in SAS Studio software version 3.81 using SAS survey procedures (SAS Institute). All *P* values were from 2-sided tests and results were statistically significant at *P* < .05.

## Results

The total cohort of respondents eligible for CCS was 5399. We were unable to include cisgender women aged 21 to 24 years because the survey instrument grouped these observations with those aged 18 to 20 years, who are ineligible for CCS.^1^ Observations missing sexual orientation (112 participants), Papanicolaou test outcome (45 participants), or both (15 participants) were removed. In logistic regression models, observations were removed that were missing at least 1 variable.

The analytical sample included 5167 cisgender women aged 25 to 64 years with no history of hysterectomy. More than 90% of the sample (4720 participants) identified as heterosexual, and 447 identified as LGB. Among LGB cisgender women, 318 (71.14%) reported up-to-date CCS compared with 3632 (76.95%) heterosexual cisgender women. The sample of LGB cisgender women skewed significantly younger than the sample of heterosexual cisgender women. A total of 358 LGB participants (80.09%) reported having a PCP compared with 4048 (85.76%) heterosexual participants (Table 1).

**Table 1.** Descriptive Characteristics of Participants by Sexual Orientation

Characteristic	Participants, No. (%)			*P* value^a^
	Total (N = 5167)	LGB (n = 447 [8.65%])	Heterosexual (n = 4720 [91.35%])	
Up-to-date cervical cancer screening				
Yes	3950	318 (71.14)	3632 (76.95)	.01
No	1217	129 (28.86)	1088 (23.05)	
Age group, y				
25-29	814	143 (31.99)	671 (12.44)	<.001
30-44	2318	233 (49.89)	2095 (44.39)	
45-64	2035	81 (18.12)	1954 (41.40)	
Income level, % of Federal Poverty Level				
<199	1842	149 (33.33)	1693 (35.87)	.78
200-399	1033	108 (24.16)	925 (19.60)	
≥400	2254	190 (42.51)	2064 (43.73)	
Missing	38	0	38 (0.81)	
Race				
Asian American, Pacific Islander or Native Hawaiian	348	27 (6.04)	321 (6.80)	.48
Black or African American	1474	103 (23.04)	1371 (29.05)	
White	2757	265 (59.28)	2492 (52.80)	
Some other race^b^	572	52 (11.63)	520 (11.02)	
Missing	16	0	16 (0.34)	
Hispanic/Latina				
Yes	1188	91 (20.36)	1097 (23.24)	.18
No	3962	353 (78.97)	3609 (76.46)	
Missing	17	3 (0.67)	14 (0.30)	
Has a primary care practitioner				
Yes	4406	358 (80.09)	4048 (85.76)	<.001
No	754	89 (19.91)	665 (14.09)	
Missing	7	0	7 (0.15)	
Insured				
Yes	4600	393 (87.92)	4207 (89.13)	.39
No	550	53 (11.86)	497 (10.53)	
Missing	17	1 (0.22)	16 (0.34)	

Abbreviation: LGB, lesbian, gay, and/or bisexual.

^a^ *P* values were calculated with χ^2^ tests.

^b^ Some other race means that the participant self-identified as “some other race” or American Indian or Alaska Native.

Sixty-one percent of Asian American, Pacific Islander or Native Hawaiian cisgender women (213 of 348 women) were up-to-date on CCS, compared with 1144 of 1474 Black or African American cisgender women (78%), 2187 of 2757 White cisgender women (79%), and 398 of 572 cisgender women of some other race (69%). Seventy-four percent of Hispanic/Latina cisgender women (883 of 1188 women) were up-to-date on CCS compared with 77% (3056 of 3962 women) of their non-Hispanic/Latina counterparts.

In stratified analysis of 5167 participants, among Asian American, Pacific Islander or Native Hawaiian cisgender women, the prevalence of up-to-date CCS was 11% lower for LGB cisgender women compared with heterosexual individuals (PR, 0.89; 95% CI, 0.87-0.91). Among Black or African American cisgender women, this prevalence was 15% lower (PR, 0.85; 95% CI, 0.84-0.85), among White cisgender women it was 5% lower (PR, 0.95; 95% CI, 0.95-0.96), and among cisgender women of some other race it was 13% lower (PR, 0.87; 95% CI, 0.86-0.88). Among Hispanic/Latina cisgender women, this prevalence was 5% lower (PR, 0.95; 95% CI, 0.94-0.96), vs 11% lower among their non-Hispanic/Latina counterparts (PR, 0.89; 95% CI, 0.89-0.89). Among cisgender women without a PCP, this prevalence was 28% lower for LGB individuals (PR, 0.72; 95% CI, 0.71-0.73), compared with 6% lower among those with a PCP (PR, 0.94; 95% CI, 0.94-0.95) (Table 2).

**Table 2.** Stratified PRs of Up-to-Date Cervical Cancer Screening, Lesbian, Gay, or Bisexual vs Heterosexual Participants (N = 5167)

Variable	Weighted stratum-specific PR (95% CI)	Breslow-Day *P* value
Age group, y		
25-29	0.93 (0.93-0.94)	<.001
30-44	0.87 (0.87-0.88)	
45-64	0.89 (0.88-0.90)	
Income level, % of Federal Poverty Level		
<199	0.89 (0.88-0.90)	<.001
200-399	0.98 (0.97-0.98)	
≥400	0.90 (0.89-0.90)	
Race		
Asian American, Pacific Islander or Native Hawaiian	0.89 (0.87-0.91)	<.001
Black or African American	0.85 (0.84-0.85)	
White	0.95 (0.95-0.96)	
Some other race^a^	0.87 (0.86-0.87)	
Hispanic/Latina		
Yes	0.95 (0.94-0.96)	<.001
No	0.89 (0.89-0.89)	
Has a primary care practitioner		
Yes	0.94 (0.94-0.95)	<.001
No	0.72 (0.71-0.73)	
Insured		
Yes	0.91 (0.90-0.91)	<.001
No	0.90 (0.89-0.91)	

Abbreviation: PR, prevalence ratio.

^a^ Some other race means the participant self-identified as “some other race” or American Indian or Alaska Native.

In multivariable analysis, the model was adjusted for age and included income level, race, having a PCP, and insurance status. Compared with heterosexual individuals, LGB sexual orientation was associated with a 10% lower prevalence of up-to-date CCS (PR, 0.90; 95% CI, 0.82-1.00). Income level, Asian American, Pacific Islander or Native Hawaiian race, having a PCP (PR, 1.43; 95% CI, 1.29-1.59), and having health insurance were significant independent variables associated with up-to-date screening (Table 3).

**Table 3.** PRs of Up-to-Date Cervical Cancer Screening (n = 5081)^a^

Variable	PR (95% CI)	
	Unadjusted	Age adjusted
Sexual orientation		
Lesbian, gay, and/or bisexual	0.92 (0.83-1.02)	0.90 (0.82-1.00)^b^
Heterosexual	1 [Reference]	1 [Reference]
Income level, % of Federal Poverty Level		
<199	0.82 (0.77-0.87)^c^	0.82 (0.77-0.87)^c^
200-399	0.92 (0.86-0.97)^d^	0.92 (0.87-0.98)^d^
≥400	1 [Reference]	1 [Reference]
Race		
Asian American, Pacific Islander or Native Hawaiian	0.81 (0.72-0.90)^c^	0.80 (0.71-0.90)^c^
Black or African American	1.05 (0.99-1.12)	1.06 (1.00-1.13)
Some other race^e^	0.98 (0.89-1.07)	0.98 (0.89-1.07)
White	1 [Reference]	1 [Reference]
Has a primary care practitioner		
Yes	1.42 (1.28-1.57)^c^	1.43 (1.29-1.59)^c^
No	1 [Reference]	1 [Reference]
Insured		
Yes	1.14 (1.03-1.27)^d^	1.15 (1.03-1.27)^d^
No	1 [Reference]	1 [Reference]

Abbreviation: PR, prevalence ratio.

^a^ Table data were generated from multivariable logistic regression models.

^b^ χ^2^ *P* value < .05.

^c^ χ^2^ *P* value < .001.

^d^ χ^2^ *P* value < .01.

^e^ Some other race means the participant self-identified as “some other race” or American Indian or Alaska Native.

In further interaction analysis, there was no significant multiplicative interaction for any covariate, consistent with the regression model. However, the interaction between LGB sexual orientation and having a PCP is reported because of its importance in the Health Equity Promotion Model and significance on an additive scale.^9^ LGB cisgender women with a PCP were 93% more likely to be up-to-date on CCS compared with LGB cisgender women without a PCP (PR, 1.93; 95% CI, 1.37-2.72). In addition, heterosexual cisgender women with a PCP were 47% more likely to be up-to-date on CCS compared with heterosexual cisgender women without a PCP (PR, 1.47; 95% CI, 1.31-1.64) (Table 4). The relative excess proportion of up-to-date CCS due to interaction was 0.18 (95% CI, 0.13-0.23).

**Table 4.** Additive Interaction Effect of Having a PCP on Up-to-Date Cervical Cancer Screening by Sexual Orientation (n = 5107)

Variable	PR (95% CI)^a^	
	Heterosexual	Lesbian, gay, and/or bisexual
No PCP	1 [Reference]	1 [Reference]
Has PCP	1.47 (1.31-1.64)	1.93 (1.37-2.72)
*P* value	<.001	<.001

Abbreviations: PCP, primary care practitioner; PR, prevalence ratio.

^a^ PRs were weighted and adjusted for age. The measure of interaction on an additive scale (ie, the relative excess proportion due to interaction) was 0.18 (95% CI, 0.13-0.23). The measure of interaction on multiplicative scale (ie, the ratio of PRs) was  1.32 (95% CI, 0.01-153.29).

## Discussion

This cross-sectional study found that LGB cisgender women are less likely to be up-to-date on CCS than heterosexual cisgender women. Also, having a PCP is an important factor associated with up-to-date CCS in Chicago—regardless of sexual orientation—but the association of having a PCP is greater for LGB individuals. Although LGB populations are less likely to receive screening than heterosexual populations, this disparity may be reduced with improved health care access and better pathways for LGB patients to establish care with a PCP.^15^ Interestingly, despite Black cisgender women having one of the highest rates of up-to-date CCS in this study, Black LGB cisgender women showed the greatest disparity in up-to-date CCS compared with their heterosexual counterparts. Consistent with current literature, better practitioner education about the experience of LGB women, and LGB women of color in particular, is needed to improve the CCS experience for these patients and thereby increase rates of primary care visits and regular CCS.^16^

### Limitations

There are limitations to this study. First, the incidence of CCS in the studied population cannot be determined. All prevalence estimates may have been impacted by selection and nonresponse bias, although they were partially corrected through weighting. A causal or temporal relationship between sexual orientation, covariates, and up-to-date CCS cannot be established. As such, these results do not reflect individual experiences with CCS. In addition, the effects of social desirability, random bias, and recall bias should be considered because most variables studied required self-report. This may be especially true for the report of the most recent Papanicolaou test.^17^ Furthermore, there were limitations in the survey design. The survey defined up-to-date CCS as having had a Papanicolaou test within 3 years; however, women who cotest with an HPV DNA test are clinically up-to-date on CCS for up to 5 years. The survey may have underestimated up-to-date CCS for individuals older than 30 years old for whom cotesting is commonly recommended and used.^1,18^ Furthermore, we were unable to include survey respondents aged 21 to 24 years because they were grouped in a broader 18 to 24 years age category and CCS is not recommended for those younger than 21 years.^1^

## Conclusions

The findings of this cross-sectional study suggest that more expansive health care access and establishment of care between LGB individuals and PCPs may improve CCS utilization for this population. These results may be generalizable to Chicago and similar diverse metropolitan areas. Our health system and subsequent research have a responsibility to explore ways to establish and maintain longitudinal relationships between LGB patients and their PCPs. Qualitative research methods and culturally appropriate interventions are recommended to better understand and address disparities in screening between LGB and heterosexual cisgender women in Chicago and the US.

## Notes

Supplement 1. **eAppendix.** Survey

Supplement 2.
Data Sharing Statement
