# Supplementary material for: Sexual Orientation and Cervical Cancer Screening Among Cisgender Women
Source: JAMA Netw Open. 2024 May 6;7(5):e248886. doi: 10.1001/jamanetworkopen.2024.8886 (PMC11074807; doi:10.1001/jamanetworkopen.2024.8886)
Supplement: Supplement 1. — eAppendix. Survey [file jamanetwopen-e248886-s001.pdf]

Supplemental Online Content

Baumann K, Matzke H, Peterson CE, et al. Sexual orientation and cervical cancer screening among cisgender women. *JAMA Netw Open*. 2024;7(5):e248886. doi:10.1001/jamanetworkopen.2024.8886

eAppendix. Survey

This supplemental material has been provided by the authors to give readers additional information about their work.

© 2024 Baumann K et al. *JAMA Network Open.*

INTRODUCTION

Thank you for completing this survey! It is being conducted on behalf of the Chicago Department of Public Health (CDPH). Information you provide will help CDPH learn about the health of people in *your* neighborhood and how to make things better. For example, your information will help CDPH create programs to reduce smoking, improve access to health services, and ensure all Chicagoans can get healthy food.

Completing this survey takes about 20 minutes, and any information you provide will be confidential. Participation is voluntary.

If you have questions or concerns about this survey, please visit www.HealthyChicagoSurvey.org, call us toll-free at 1-866-784-7723 or email us at HealthyChicagoSurvey@rti.org.

We'll ask questions about your health and things that can influence your health, like your neighborhood and whether you have access to health services.

INSTRUCTIONS

- **This survey should be completed by the adult (must be 18 years of age or older) in the household who will have the next birthday. This helps to ensure a representative study of Chicago residents.**
- **Answer all of the questions by completely filling in the circle to the left of your answer, like this:**

  ● Yes
  ○ No

- **You are sometimes told to skip over some questions in this survey. When this happens, you will see an arrow with a note that tells you which question to answer next, like this:**

  12. Has a doctor, nurse, or other health professional ever told you that you had…asthma?
  ○ Yes
  ○ No → ***Go to #13***

  12a. Do you still have asthma?
  ○ Yes
  ○ No

  In this example, if you answer "Yes" to Question 12, you should continue to Question 12a.

  If you answer "No" to Question 12, you should continue to Question 13.

- **Use a black or blue pen, if available.**

© 2024 Baumann K et al. *JAMA Network Open.*

START HERE

GENERAL HEALTH

1. Would you say that in general your health is…?

- ◯ Excellent
- ◯ Very good
- ◯ Good
- ◯ Fair
- ◯ Poor

2. Do you have at least one person you think of as your personal doctor or health care provider?

- ◯ Yes
- ◯ No

3. A routine checkup is a general physical exam, not an exam for a specific injury, illness, or condition. About how long has it been since you last visited a doctor or health care provider for a routine checkup?

- ◯ Within the past year
- ◯ One or more years ago
- ◯ Never

4. In general, how satisfied are you with the health care you received in the past 12 months?

- ◯ Very satisfied
- ◯ Somewhat satisfied
- ◯ Not at all satisfied
- ◯ I did not receive any health care in the past 12 months

5. How long has it been since you had your teeth cleaned by a dentist or dental hygienist?

- ◯ 6 months or less
- ◯ More than 6 months, but not more than one year ago
- ◯ More than one year ago
- ◯ Never

6. Do you have any kind of health care coverage, including health insurance, prepaid plans such as HMOs, government plans such as Medicare, or Indian Health Services?

- ◯ Yes
- ◯ No → ***Go to #7***

6a. What is the <u>primary</u> source of your health care coverage?

- ◯ A plan purchased through an employer or union (includes plans purchased through another person's employer)
- ◯ A plan that you or another family member buys on your own
- ◯ Medicare
- ◯ Medicaid or other state program
- ◯ TRICARE (formerly CHAMPUS), VA, or Military
- ◯ Alaska Native, Indian Health Service, Tribal Health Services
- ◯ Some other source

6b. In the past 12 months, how often was it easy to get the care, tests or treatment you thought you needed through your health plan?

- ◯ Never
- ◯ Sometimes
- ◯ Usually
- ◯ Always
- ◯ I didn't need care, tests, or treatment in the past 12 months

© 2024 Baumann K et al. *JAMA Network Open.*

CHRONIC HEALTH CONDITIONS

The next questions ask whether a doctor, nurse, or other health professional ever told you that you had any of the following health conditions.

By "other health professional", we mean a nurse practitioner, a physician's assistant, or some other licensed health professional.

7. Has a doctor, nurse, or other health professional ever told you that you had…high blood pressure?

- ◯ Yes
- ◯ Yes, but only while I was pregnant
- ◯ No

8. Has a doctor, nurse, or other health professional ever told you that you had…high cholesterol?

- ◯ Yes
- ◯ No

9. Has a doctor, nurse, or other health professional ever told you that you had…angina or coronary heart disease?

- ◯ Yes
- ◯ No

10. Has a doctor, nurse, or other health professional ever told you that you had…diabetes?

- ◯ Yes
- ◯ Yes, but only while I was pregnant
- ◯ No

11. Has a doctor, nurse, or other health professional ever told you that you had…chronic obstructive pulmonary disease, COPD, emphysema, or chronic bronchitis?

- ◯ Yes
- ◯ No

12. Has a doctor, nurse, or other health professional ever told you that you had…asthma?

- ◯ Yes
- ◯ No → ***Go to #13***

12a. Do you still have asthma?

- ◯ Yes
- ◯ No

TOBACCO USE

13. Have you smoked at least 100 cigarettes (approximately 5 packs) in your entire life?

- ◯ Yes
- ◯ No → ***Go to #14***

13a. Do you now smoke cigarettes every day, some days, or not at all?

- ◯ Every day] ***Go to #13c***
- ◯ Some days] ***Go to #13c***
- ◯ Not at all → ***Go to #13b***

13b. How long has it been since you last smoked a cigarette, even one or two puffs?

- ◯ Less than 1 year ago
- ◯ More than 1 year but less than 5 years ago
- ◯ More than 5 years but less than 10 years ago
- ◯ 10 years or more
- ◯ Never smoked regularly

] ***Go to #14***

© 2024 Baumann K et al. *JAMA Network Open.*

13c. Currently, when you smoke cigarettes, how often do you smoke menthol cigarettes?

- ◯ All of the time
- ◯ Most of the time
- ◯ Some of the time
- ◯ None of the time

13d. During the past 12 months, have you stopped smoking for one day or longer because you were trying to quit smoking?

- ◯ Yes
- ◯ No

14. Have you <u>ever</u> tried an e-cigarette or vaped, even one or two puffs? This would include products like JUUL, Blu, and NJOY.

Do not include using electronic vaping products with marijuana or cannabis.

- ◯ Yes
- ◯ No → ***Go to #15***

14a. How often do you use e-cigarettes or vape now?

- ◯ Every day
- ◯ Some days
- ◯ Not at all

CANNABIS USE

The next questions are about marijuana or cannabis, which became legal in Illinois on January 1, 2020. These questions do not refer to CBD or other non-THC products. Your answers are strictly confidential.

15. Have you ever, even once, tried marijuana or cannabis?

- ◯ Yes
- ◯ No → ***Go to #16***

15a. Have you used marijuana or cannabis during the past 30 days?

- ◯ Yes
- ◯ No → ***Go to #16***

15b. During the past 30 days, on how many days did you use marijuana or cannabis?

☐☐ Days

15c. On days that you used marijuana, how many times per day did you use on average?

☐ Times per day

15d. When you used marijuana or cannabis during the past 30 days, was it usually for…?

- ◯ Medical reasons (like to treat or decrease symptoms or health conditions)
- ◯ Non-medical reasons (like to have fun or fit in)
- ◯ Both medical and non-medical reasons

© 2024 Baumann K et al. *JAMA Network Open.*

15e. During the past 30 days, how did you use marijuana? Did you …?

Select Yes or No for each statement.

	Yes	No
Smoke it (like in a joint, bong, pipe or blunt)	◯	◯
Eat it (like in brownies, cakes, cookies or candy)	◯	◯
Drink it (like in tea, cola or alcohol)	◯	◯
Vape it (like in an e-cigarette-like vaporizer)	◯	◯
Dab it (like using butane hash oil, wax or concentrates)	◯	◯
Other (please specify):	◯	◯

DIET & PHYSICAL ACTIVITY

16. How many total servings of <u>fruit</u> did you eat yesterday?

A serving would equal one medium apple or a handful of grapes. Please think about all forms of fruits including cooked or raw, fresh, frozen, or canned.

Please think about all meals, snacks, and food consumed at home and away from home. If none, enter 00.

☐☐ Servings

17. How many total servings of <u>vegetables</u> did you eat yesterday?

A serving would equal a handful of broccoli or a cup of carrots. Please think about all forms of vegetables including cooked or raw, fresh, frozen, or canned.

Please think about all meals, snacks, and food consumed at home and away from home. If none, enter 00.

☐☐ Servings

18. How easy or difficult is it for you to get fresh produce (fruits and vegetables)?

- ◯ Very difficult
- ◯ Somewhat difficult
- ◯ Somewhat easy
- ◯ Very easy

19. How true is the following statement: "In the past 12 months, we worried whether our food would run out before we got money to buy more."

- ◯ Often true
- ◯ Sometimes true
- ◯ Never true

© 2024 Baumann K et al. *JAMA Network Open.*

20. **During the past 30 days, how many regular soda or pop or other sweetened drinks like sweetened iced tea, sports drinks, fruit punch, or other fruit-flavored drinks have you had?**

Do not include diet soda, sugar free drinks, or 100% juice. If none, enter 00.

☐☐ Drinks

Select the period of time (per day/week/month):

- ◯ Drinks per day
- ◯ Drinks per week
- ◯ Drinks per month

21. **During the past month, other than your regular job, did you participate in any physical activities or exercises such as running, dance, playing basketball, taking an exercise class, gardening, or walking for exercise?**

- ◯ Yes
- ◯ No

22. **In the past 12 months, how often did you or someone in your household use the parks, playgrounds and sport fields in your neighborhood?**

- ◯ Once a week or more
- ◯ Several times a month
- ◯ At least once a month
- ◯ A few times a year
- ◯ Never

23. **In the past 12 months, how often have you ridden a bicycle in Chicago?**

- ◯ Once a week or more
- ◯ Several times a month
- ◯ At least once a month
- ◯ A few times a year
- ◯ Never
- ◯ I am not physically able to ride a bike

24. **During the past 7 days, did you ever walk or use a wheelchair to get to and from places such as work, shopping, or other activities?**

- ◯ Yes
- ◯ No
- ◯ I am not physically able to walk or use a wheelchair

25. **About how tall are you without shoes?**

☐ Feet ☐☐ Inches

26. **About how much do you weigh without shoes?**

If you are currently pregnant, how much did you weigh before your pregnancy?

☐☐☐ Pounds

© 2024 Baumann K et al. *JAMA Network Open.*

ALCOHOL & PRESCRIPTION DRUGS

27. During the past 30 days, how many days did you have at least one drink of any alcoholic beverage?

One drink is equivalent to a 12-ounce beer, a 5-ounce glass of wine, or a drink with one shot of liquor. A 40-ounce beer would count as 3 drinks, or a cocktail drink with 2 shots would count as 2 drinks. If none, enter 00.

☐☐ Days

28a. [*FOR MEN ONLY*] Considering all types of alcoholic beverages, how many times during the past 30 days did you have 5 or more drinks on one occasion?

One drink is equivalent to a 12-ounce beer, a 5-ounce glass of wine, or a drink with one shot of liquor. A 40-ounce beer would count as 3 drinks, or a cocktail drink with 2 shots would count as 2 drinks. If none, enter 00.

☐☐ Times

28b. [*FOR WOMEN ONLY*] Considering all types of alcoholic beverages, how many times during the past 30 days did you have 4 or more drinks on one occasion?

One drink is equivalent to a 12-ounce beer, a 5-ounce glass of wine, or a drink with one shot of liquor. A 40-ounce beer would count as 3 drinks, or a cocktail drink with 2 shots would count as 2 drinks. If none, enter 00.

☐☐ Times

The next few questions are about medications that require a prescription. Do not include 'over the counter' medications such as aspirin, Tylenol, or Advil which can be bought in drug stores without a doctor's prescription. Your answers are strictly confidential.

29. In the past 12 months, have you ever taken a prescription pain reliever such as oxycodone or hydrocodone that was prescribed to you?

- ○ Yes
- ○ No → ***Go to #30***

29a. When you took prescription pain relievers in the past 12 months, did you ever, even once, take more than was prescribed for you? This includes taking a higher dosage or taking it more often than directed.

- ○ Yes
- ○ No

30. In the past 12 months, have you ever, even once, taken a prescription pain reliever such as oxycodone or hydrocodone that was not prescribed for you?

- ○ Yes
- ○ No

© 2024 Baumann K et al. *JAMA Network Open.*

CANCER SCREENING

31. What is your gender?

- ◯ Male → ***Go to #35***
- ◯ Female
- ◯ Prefer to self-describe

32. A mammogram is an x-ray of each breast to look for breast cancer. Have you ever had a mammogram?

- ◯ Yes
- ◯ No → ***Go to #33***

32a. How long has it been since you had your last mammogram?

- ◯ Less than 12 months ago
- ◯ At least 1 year ago but less than 2 years ago
- ◯ At least 2 years ago but less than 3 years ago
- ◯ At least 3 years ago but less than 5 years ago
- ◯ 5 or more years ago

33. A Pap test is a test for cancer of the cervix. Have you ever had a Pap test?

- ◯ Yes
- ◯ No → ***Go to #34***

33a. How long has it been since your last Pap test?

- ◯ Less than 12 months ago
- ◯ At least 1 year ago but less than 2 years ago
- ◯ At least 2 years ago but less than 3 years ago
- ◯ At least 3 years ago but less than 5 years ago
- ◯ 5 or more years ago

34. Have you had a hysterectomy?

- ◯ Yes
- ◯ No

35. A blood stool test is a test that may use a special kit at home to determine whether the stool contains blood. Have you ever had this test using a home kit?

- ◯ Yes
- ◯ No → ***Go to #36***

35a. How long has it been since you had your last blood stool test using a home kit?

- ◯ Less than 12 months ago
- ◯ At least 1 year ago but less than 2 years ago
- ◯ At least 2 years ago but less than 3 years ago
- ◯ At least 3 years ago but less than 5 years ago
- ◯ 5 or more years ago

© 2024 Baumann K et al. *JAMA Network Open.*

36. Sigmoidoscopy and colonoscopy are exams in which a tube is inserted in the rectum to view the colon for signs of cancer or other health problems.

For a *__sigmoidoscopy__**, a flexible tube is inserted into the rectum to look for problems.*

A *__colonoscopy__* *is similar, but uses a longer tube, and you are usually given medication through a needle in your arm to make you sleepy and told to have someone else drive you home after the test.*

Have you ever had either of these exams?

- ◯ Yes
- ◯ No → ***Go to #37***

36a. Was your __most recent__ exam a sigmoidoscopy or a colonoscopy?

- ◯ Sigmoidoscopy
- ◯ Colonoscopy

36b. How long has it been since you had your last sigmoidoscopy or colonoscopy?

- ◯ Less than 12 months ago
- ◯ At least 1 year ago but less than 2 years ago
- ◯ At least 2 years ago but less than 3 years ago
- ◯ At least 3 years ago but less than 5 years ago
- ◯ 5 or more years ago

MENTAL HEALTH

During the past 30 days, how often did you feel…

37. …__nervous__?

- ◯ All of the time
- ◯ Most of the time
- ◯ Some of the time
- ◯ A little of the time
- ◯ None of the time

38. …__hopeless__?

- ◯ All of the time
- ◯ Most of the time
- ◯ Some of the time
- ◯ A little of the time
- ◯ None of the time

39. …__restless or fidgety__?

- ◯ All of the time
- ◯ Most of the time
- ◯ Some of the time
- ◯ A little of the time
- ◯ None of the time

40. …__so depressed that nothing could cheer you up__?

- ◯ All of the time
- ◯ Most of the time
- ◯ Some of the time
- ◯ A little of the time
- ◯ None of the time

© 2024 Baumann K et al. *JAMA Network Open.*

During the past 30 days, how often did you feel…

41. …<u>everything was an effort</u>?

- ◯ All of the time
- ◯ Most of the time
- ◯ Some of the time
- ◯ A little of the time
- ◯ None of the time

42. …<u>worthless</u>?

- ◯ All of the time
- ◯ Most of the time
- ◯ Some of the time
- ◯ A little of the time
- ◯ None of the time

43. How often do you feel lonely?

- ◯ Almost always
- ◯ Often
- ◯ Sometimes
- ◯ Hardly ever
- ◯ Never

44. Are you now taking medicine or receiving treatment from a doctor or other health professional for any type of mental health condition or emotional problem?

- ◯ Yes
- ◯ No

45. During the past 12 months, was there any time when you needed mental health treatment or counseling for yourself but didn't get it?

- ◯ Yes
- ◯ No → *Go to #46*

45a. Was the following a reason why you did not get the mental health treatment or counseling you needed?

Select Yes or No for each statement.

	Yes	No
You couldn't afford the cost	◯	◯
You were concerned that getting mental health treatment or counseling might cause your neighbors or community to have a negative opinion of you	◯	◯
You were concerned that getting mental health treatment or counseling might have a negative effect on your job	◯	◯
Your health insurance does not cover any mental health treatment or counseling	◯	◯
Your health insurance does not pay enough for mental health treatment or counseling	◯	◯
You did not know where to go to get services	◯	◯
You were concerned that the information you gave the counselor might not be kept confidential	◯	◯
You were concerned that you might be committed to a psychiatric hospital or might have to take medicine	◯	◯
Other (please specify):	◯	◯

© 2024 Baumann K et al. *JAMA Network Open.*

46. On average, how many hours of sleep do you get in a 24-hour period?

[ ][ ] Hours [ ][ ] Minutes

FINANCIAL SECURITY

47. How often in the past 12 months would you say you were worried or stressed about having enough money to pay rent or mortgage?

- ○ Always
- ○ Usually
- ○ Sometimes
- ○ Never

48. In the past 12 months, how often did you put off paying for food to pay for <u>housing</u>?

- ○ Often
- ○ Sometimes
- ○ Rarely
- ○ Never

49. In the past 12 months, how often did you put off paying for food to pay <u>your utility bill</u>?

- ○ Often
- ○ Sometimes
- ○ Rarely
- ○ Never

50. In the past 12 months, how often did you put off paying for food to pay for <u>medicine or health care</u>?

- ○ Often
- ○ Sometimes
- ○ Rarely
- ○ Never

51. In the past 12 months, how often did you put off paying for food to pay for <u>gas or transportation</u>?

- ○ Often
- ○ Sometimes
- ○ Rarely
- ○ Never

52. Suppose that you have an emergency expense that costs $400. Based on your current financial situation, would you…?

Select Yes or No for each statement.

	Yes	No
Put it on your credit card and pay it off in full at the next statement	○	○
Put it on your credit card and pay it off over time	○	○
Pay with the money currently in your checking/savings account or with cash	○	○
Use money from a bank loan or line of credit	○	○
Borrow from a friend or family member	○	○
Use a payday loan, deposit advance or overdraft	○	○
Sell something	○	○
Not be able to pay for the expense right now	○	○
Other (please specify): [ ]	○	○

53. Do you or anyone in your household currently have a checking or savings account?

- ○ Yes
- ○ No

© 2024 Baumann K et al. *JAMA Network Open.*

YOUR NEIGHBORHOOD

54. How long have you lived in your neighborhood?

- ◯ Less than one year
- ◯ At least 1 year, but less than 5 years
- ◯ At least 5 years, but less than 10 years
- ◯ At least 10 years, but less than 20 years
- ◯ 20 years or longer

55. People move for many different reasons. Thinking of your most recent move, did you move…?

Select Yes or No for each statement.

	Yes	No
To be closer to work or school	◯	◯
To be closer to family or friends	◯	◯
For better quality neighborhood or schools	◯	◯
Because you received an eviction notice	◯	◯
Because your previous home or apartment was foreclosed	◯	◯
Your rent increased at previous home or apartment	◯	◯
Your landlord would not fix things at previous home or apartment	◯	◯
To save money	◯	◯
To relocate to a new city	◯	◯
Because your family status changed (e.g. marriage, divorce, children, adult child moved out)	◯	◯
For a better quality or larger home	◯	◯
Because you bought a home	◯	◯
Other (please specify):	◯	◯

Thinking about your current neighborhood, to what extent do you agree or disagree with the following statements:

56. The sidewalks in my neighborhood are well-maintained (paved, even and not a lot of cracks).

- ◯ Strongly agree
- ◯ Agree
- ◯ Neither agree nor disagree
- ◯ Disagree
- ◯ Strongly disagree

57. It is easy to walk, scoot, or roll to a transit stop (bus, train) from my home.

- ◯ Strongly agree
- ◯ Agree
- ◯ Neither agree nor disagree
- ◯ Disagree
- ◯ Strongly disagree

58. My neighborhood is generally free from litter.

- ◯ Strongly agree
- ◯ Agree
- ◯ Neither agree nor disagree
- ◯ Disagree
- ◯ Strongly disagree

59. There are many interesting things to look at while walking, scooting, or rolling in my neighborhood.

- ◯ Strongly agree
- ◯ Agree
- ◯ Neither agree nor disagree
- ◯ Disagree
- ◯ Strongly disagree

© 2024 Baumann K et al. *JAMA Network Open.*

60. Do you feel safe in your neighborhood?

- ◯ Yes, all of the time
- ◯ Yes, most of the time
- ◯ Sometimes
- ◯ No, mostly not

61. In your neighborhood, how often does violence occur?

- ◯ Every day
- ◯ At least every week
- ◯ At least every month
- ◯ Every few months
- ◯ Once a year or so
- ◯ Not at all

62. Since age 18, have you ever been arrested, booked, or charged for breaking the law?

- ◯ Yes
- ◯ No

63. Would you say that you really feel part of your neighborhood?

- ◯ Strongly agree
- ◯ Agree
- ◯ Neither agree nor disagree
- ◯ Disagree
- ◯ Strongly disagree

64. About how many people in your neighborhood do you know well enough to ask for help if you needed it? *If none, enter 00.*

[ ][ ] People

65. Would you say that you regularly stop and talk with people in your neighborhood?

- ◯ Strongly agree
- ◯ Agree
- ◯ Neither agree nor disagree
- ◯ Disagree
- ◯ Strongly disagree

66. To what extent do you feel like you and your neighbors have the ability to impact your community?

- ◯ A great extent
- ◯ Somewhat
- ◯ A little
- ◯ Not at all

67. To what extent do you trust local government to do what's right for your community?

- ◯ A great extent
- ◯ Somewhat
- ◯ A little
- ◯ Not at all

68. To what extent do you trust your law enforcement agency?

- ◯ A great extent
- ◯ Somewhat
- ◯ A little
- ◯ Not at all

© 2024 Baumann K et al. *JAMA Network Open.*

69. Thinking about the past 12 months, have you done any of the following…?

Select Yes or No for each statement.

	Yes	No
Attended a neighborhood meeting about a local issue	◯	◯
Voted in the last election	◯	◯
Visited a museum	◯	◯
Visited a Chicago Public Library location	◯	◯
Attended a community event, block party, parade or festival	◯	◯
Participated in art or cultural activities	◯	◯

CHILDREN AND TEENS

How big of a problem do you feel the following issues are for children and teens across the city of Chicago?

70. …alcohol abuse by youth?

- ◯ A big problem
- ◯ Somewhat of a problem
- ◯ Not a problem
- ◯ Don’t know/not sure

71. …childhood asthma?

- ◯ A big problem
- ◯ Somewhat of a problem
- ◯ Not a problem
- ◯ Don’t know/not sure

72. …child abuse and neglect?

- ◯ A big problem
- ◯ Somewhat of a problem
- ◯ Not a problem
- ◯ Don’t know/not sure

73. …depression among children and teens?

- ◯ A big problem
- ◯ Somewhat of a problem
- ◯ Not a problem
- ◯ Don’t know/not sure

74. …drug abuse by youth?

- ◯ A big problem
- ◯ Somewhat of a problem
- ◯ Not a problem
- ◯ Don’t know/not sure

75. …infant mortality?

- ◯ A big problem
- ◯ Somewhat of a problem
- ◯ Not a problem
- ◯ Don’t know/not sure

76. …injuries from accidents among children and teens?

- ◯ A big problem
- ◯ Somewhat of a problem
- ◯ Not a problem
- ◯ Don’t know/not sure

77. …childhood obesity?

- ◯ A big problem
- ◯ Somewhat of a problem
- ◯ Not a problem
- ◯ Don’t know/not sure

© 2024 Baumann K et al. *JAMA Network Open.*

How big of a problem do you feel the following issues are for children and teens across the city of Chicago?

78. …parent's health problems affecting their children?

- A big problem
- Somewhat of a problem
- Not a problem
- Don't know/not sure

79. …smoking and tobacco use by youth, including vaping or using e-cigarettes?

- A big problem
- Somewhat of a problem
- Not a problem
- Don't know/not sure

80. …stress among children and teens?

- A big problem
- Somewhat of a problem
- Not a problem
- Don't know/not sure

81. …suicide among children and teens?

- A big problem
- Somewhat of a problem
- Not a problem
- Don't know/not sure

82. …teen pregnancy?

- A big problem
- Somewhat of a problem
- Not a problem
- Don't know/not sure

83. …bullying, including cyberbullying?

- A big problem
- Somewhat of a problem
- Not a problem
- Don't know/not sure

84. …discrimination and racism?

- A big problem
- Somewhat of a problem
- Not a problem
- Don't know/not sure

85. …gun-related violence in neighborhoods?

- A big problem
- Somewhat of a problem
- Not a problem
- Don't know/not sure

86. …hunger?

- A big problem
- Somewhat of a problem
- Not a problem
- Don't know/not sure

87. …lack of adult supervision and involvement for children and teens?

- A big problem
- Somewhat of a problem
- Not a problem
- Don't know/not sure

© 2024 Baumann K et al. *JAMA Network Open.*

How big of a problem do you feel the following issues are for children and teens across the city of Chicago?

88. …<u>not enough job opportunities for parents</u>?

- ◯ A big problem
- ◯ Somewhat of a problem
- ◯ Not a problem
- ◯ Don't know/not sure

89. …<u>not enough job opportunities for teens and young adults</u>?

- ◯ A big problem
- ◯ Somewhat of a problem
- ◯ Not a problem
- ◯ Don't know/not sure

90. …<u>poverty</u>?

- ◯ A big problem
- ◯ Somewhat of a problem
- ◯ Not a problem
- ◯ Don't know/not sure

91. …<u>social media</u>?

- ◯ A big problem
- ◯ Somewhat of a problem
- ◯ Not a problem
- ◯ Don't know/not sure

92. …<u>unsafe housing</u>?

- ◯ A big problem
- ◯ Somewhat of a problem
- ◯ Not a problem
- ◯ Don't know/not sure

93. …<u>violence in schools</u>?

- ◯ A big problem
- ◯ Somewhat of a problem
- ◯ Not a problem
- ◯ Don't know/not sure

94. …<u>worse health for children of color than for white children, also known as racial inequalities</u>?

- ◯ A big problem
- ◯ Somewhat of a problem
- ◯ Not a problem
- ◯ Don't know/not sure

CORONAVIRUS & COVID-19

95. Have you or someone in your household experienced any of the following because of coronavirus or COVID-19…?

Select Yes or No for each statement.

	Yes	No
Having to spend at least one night in a hospital or quarantine facility	◯	◯
Not being able to get the food you needed	◯	◯
A loss of social connection	◯	◯
Being unable to pay your rent, mortgage or bills on time	◯	◯
Having worsened mental health or emotional problems	◯	◯
Cancelling or postponing surgery or other medical care	◯	◯
Grief from losing someone who died from COVID-19	◯	◯

© 2024 Baumann K et al. *JAMA Network Open.*

96. Have you or someone in your household been let go, had work hours reduced, or had a reduction in pay because of coronavirus or COVID-19?

- ◯ Yes
- ◯ No → ***Go to #97***

96a. To what extent was this because you or someone in your household had to take on increased childcare responsibilities?

- ◯ A great extent
- ◯ Somewhat
- ◯ A little
- ◯ Not at all

97. A new vaccine against coronavirus or COVID-19 may be available in the future. How likely would you be to get vaccinated against coronavirus or COVID-19, if a vaccine were available?

- ◯ Very likely
- ◯ Somewhat likely
- ◯ Not likely
- ◯ I'm not sure

ABOUT YOU

98. What is your age?

- ◯ 18-24
- ◯ 25-29
- ◯ 30-44
- ◯ 45-64
- ◯ 65 or older

99. What racial or ethnic group do you consider yourself to be?

____

100. Are you Hispanic or Latino/a, or of Spanish origin?

- ◯ Yes
- ◯ No → ***Go to #101***

100a. Would you say you are…?

Select Yes or No for each option.

	Yes	No
Mexican, Mexican-American, or Chicano/a	◯	◯
Puerto Rican	◯	◯
Cuban	◯	◯
Another Hispanic, Latino/a, or Spanish origin	◯	◯

© 2024 Baumann K et al. *JAMA Network Open.*

101. Which one or more of the following would you say is your race?

- ◯ White → ***Go to #102***
- ◯ Black or African American → ***Go to #102***
- ◯ American Indian or Alaska Native → ***Go to #102***
- ◯ Asian
- ◯ Native Hawaiian or Pacific Islander → ***Go to #102***
- ◯ Some other race → ***Go to #102***

101a. Would you say you are…?

Select Yes or No for each option.

	Yes	No
Asian Indian	◯	◯
Chinese	◯	◯
Filipino	◯	◯
Japanese	◯	◯
Korean	◯	◯
Vietnamese	◯	◯
Another Asian origin	◯	◯

102. Do you consider yourself to be…?

- ◯ Heterosexual or straight
- ◯ Gay or lesbian
- ◯ Bisexual
- ◯ Prefer to self-describe

103. Do you consider yourself to be transgender?

Transgender is when a person thinks of themself as a different gender than what they were assigned at birth, such as a person born female who now considers themself to be male.

- ◯ Yes
- ◯ No

104. Are you…?

- ◯ Married
- ◯ Divorced
- ◯ Widowed
- ◯ Separated
- ◯ Never married
- ◯ A member of an unmarried couple
- ◯ A member of a civil union

105. What is the highest grade or year of school you completed?

- ◯ Less than high school graduation
- ◯ High school graduation (Grade 12 or GED)
- ◯ Some college or technical school
- ◯ Associate degree
- ◯ Bachelor’s degree
- ◯ Graduate or professional degree

106. Are you currently…?

- ◯ Employed for wages
- ◯ Self-employed
- ◯ Out of work for 1 year or more
- ◯ Out of work for less than 1 year
- ◯ A Homemaker
- ◯ A Student
- ◯ Retired
- ◯ Unable to work

Go to #107

106a. Do you have more than one job?

This means more than one employer, not just multiple job sites.

- ◯ Yes
- ◯ No

© 2024 Baumann K et al. *JAMA Network Open.*

107. Do you own or rent your home?

- Own
- Rent
- Some other arrangement

108. How many people, including yourself, live in this household?

Count people who spend a majority of their time living in this household. Enter a number for each category. If none, enter 00.

- Adults, 18 years of age or older
- Children, 11-17 years old
- Children, 6-10 years old
- Children, 1-5 years old
- Children, less than 1 year old

109. [*ONLY IF #108 INCLUDES CHILDREN*]: For how many of these children are you the parent, step-parent, foster parent, or guardian?
Enter a number for each category. If none, enter 00.

- Children, 11-17 years old
- Children, 6-10 years old
- Children, 1-5 years old
- Children, less than 1 year old

110. What is your annual combined household income?

By household income, we mean the combined income from everyone living in the household including roommates or those on disability income.

Your answer is private and confidential and cannot be used to affect your benefits.

$ ___ ___ ___ , ___ ___ ___ . ___ ___

THANK YOU!

111. Please select how you would like to receive your $10.

- Electronic gift card sent by email
- Check sent by mail

112. May we contact you if we have more questions about coronavirus or COVID-19?

- Yes
- No

113. Please provide your contact information.

An email is needed to send an electronic gift card. A name is needed to send a check. All are needed to recontact you with questions about COVID-19.

First Name:

Last Name:

Email:

Phone:

Please return this questionnaire in the envelope provided or to:

Healthy Chicago Survey
c/o RTI International
0217366.000.005
PO Box 25735
Chicago, IL 60625

You will receive your $10 in three to four weeks.

© 2024 Baumann K et al. *JAMA Network Open.*
