# Supplementary material for: Sexual Orientation and Cervical Cancer Screening Among Cisgender Women
Source: JAMA Netw Open. 2024 May 6;7(5):e248886. doi: 10.1001/jamanetworkopen.2024.8886 (PMC11074807; doi:10.1001/jamanetworkopen.2024.8886)
Supplement: Supplement 2. — Data Sharing Statement [file jamanetwopen-e248886-s002.pdf]

# Data Sharing Statement

Baumann. Sexual Orientation and Cervical Cancer Screening Among Cisgender Women. *JAMA Netw Open*. Published May 06, 2024. doi:10.1001/jamanetworkopen.2024.8886

**Data**
**Data available:** No

**Additional Information**
**Explanation for why data not available:** Those interested in the Healthy Chicago Survey data should reach out directly to the Chicago Department of Public Health for access. Please reach out to Kelley Baumann for SAS data analysis files.
